# Is it ethical to prevent secondary use of stored biological samples and data derived from consenting research participants? The case of Malawi

**DOI:** 10.1186/s12910-015-0077-x

**Published:** 2015-12-02

**Authors:** Randy G. Mungwira, Wongani Nyangulu, James Misiri, Steven Iphani, Ruby Ng’ong’ola, Chawanangwa M. Chirambo, Francis Masiye, Joseph Mfutso-Bengo

**Affiliations:** College of Medicine, School of Public Health, Master of Public Health Bioethics Major Students, University of Malawi, Blantyre, Malawi; Department of Medicine, University of Cape Town, Cape Town, Republic of South Africa; College of Medicine, School of Public Health, Center for Bioethics, University of Malawi, Blantyre, Malawi

**Keywords:** Autonomy, Biological samples/data, Broad consent, Data transfer agreements, Institutional review boards, Material transfer agreements, Multi-layered/tiered consent, Research participants

## Abstract

**Background:**

This paper discusses the contentious issue of reuse of stored biological samples and data obtained from research participants in past clinical research to answer future ethical and scientifically valid research questions. Many countries have regulations and guidelines that guide the use and exportation of stored biological samples and data. However, there are variations in regulations and guidelines governing the reuse of stored biological samples and data in Sub-Saharan Africa including Malawi.

**Discussion:**

The current research ethics regulations and guidelines in Malawi do not allow indefinite storage and reuse of biological samples and data for future unspecified research. This comes even though the country has managed to answer pertinent research questions using stored biological samples and data. We acknowledge the limited technical expertise and equipment unavailable in Malawi that necessitates exportation of biological samples and data and the genuine concern raised by the regulatory authorities about the possible exploitation of biological samples and data by researchers. We also acknowledge that Malawi does not have bio-banks for storing biological samples and data for future research purposes. This creates room for possible exploitation of biological samples and data collected from research participants in primary research projects in Malawi. However, research ethics committees require completion and approval of material transfer agreements and data transfer agreements for biological samples and data collected for research purposes respectively and this requirement may partly address the concern raised by the regulatory authorities. Our concern though is that there is no such requirement for biological samples and data collected from patients for clinical or diagnostic purposes.

**Summary:**

In conclusion, we propose developing a medical data and material transfer agreement for biological samples and data collected from patients for clinical or diagnostic purposes in both public and private health facilities that may end up in research centers outside Malawi. We also propose revision of the current research ethics regulations and guidelines in Malawi in order to allow secondary use of biological samples and data collected from primary research projects as a way of maximizing the use of collected samples and data. Finally, we call for consultation of all stakeholders within the Malawi research community when regulatory authorities are developing policies that govern research in Malawi.

## Background

Clinical research may sometimes involve exportation and importation of biological samples and data which serve as the back bone of viable scientific or biomedical research. As emerging innovations in the fields of genetics, genomics and biotechnology increase, the value of biological samples and data [1, 2] creates greater demand that will lead to increased exportation of biological samples and data for technologically advanced biomedical research mostly in developed countries. One of the initiatives in this area is the H3Africa, an international collaboration of scientists which seeks to foster genetic and genomic research on diseases that are pertinent to African people with the goal of improving the health of African populations [3]. Most developed countries and some developing countries have regulations and guidelines that guide the use and exportation of stored biological samples and data in future research. However, Malawian research ethics regulations and guidelines do not allow storage and secondary use of biological samples and data in future unspecified research [4, 5]. Therefore, the aim of this paper is to address the current debate in Malawi regarding storage, use and exportation of biological samples and data and make recommendations that will protect research participants and benefit the biomedical research community in the country.

## Collection and storage of biological samples and data in the developed world

In the United States of America (US), it is allowed to collect and store biological samples and data for future research. However, there are disagreements on the issue of informed consent. According to the US federal regulations “also known as the Common rule” or the Code of Federal Regulations (CFR), research on stored biological samples and data is explicitly exempt from review by institutional review boards (IRB) [1]. This is on condition that sources cannot be “identified directly or through identifiers linked to the samples and data” [1]. Some investigators have interpreted this to mean that there is no need for informed consent so long as the biological samples and data are “anonymized” and delinked from the sources [1]. In fact, the American Society of Human Genetics (ASHG) is of the view that re-consenting individuals for research with “stored samples and data that poses only minimal risk” is not necessary [1]. Other commentators recommend a checklist of options to allow research participants during the consent process to approve or refuse different types of research [2]. This is what is referred to as multi-layered or tiered consent. The National Bioethics Advisory Commission (NBAC) in the US recommends this model of consent and specifically includes six separate choices to be provided while the National Action Plan on Breast Cancer recommends research participants to be allowed to approve research on the disease being studied and separately approve or refuse research on other diseases in what is known as broad consent [2]. In Europe, most scientists are of the view that broad consent is acceptable for biological samples and data to be used for research that was not part of the original research protocol [6].

It is important to note that these guidelines are not based on empirical research done on research participants but mostly on the interpretation held by the different bodies on the principle of respect for persons or autonomy and informed consent [1]. A study done in the US (*n* = 504) found that for biological samples and data derived from research, the majority of research participants would not require their consent for identifiable samples and data and even less than 12.1 % of the research participants would require consent for anonymized samples [1]. For clinically derived samples and data, the majority of participants (65.8 %) would require their consent for identifiable samples and data while fewer (27.3 %) would require consent for anonymized samples and data [1]. This suggests that “consent should be required for research using clinically derived, identifiable samples and data, but waived for additional research using research-derived, anonymized samples” [1]. Of note, these findings may not be generalisable as this study involved mostly elderly female Caucasian respondents with high educational levels and income [1].

## Collection and storage of biological samples and data in Sub-Saharan Africa

Most countries in Sub-Saharan Africa require researchers from both academic and research institutions to complete material transfer agreement forms (MTAs) before shipping biological samples and data collected from research participants to academic or research institutions overseas, and mostly to developed countries. In the MTA forms, the researchers are required to justify importation and exportation of biological samples and data to the institutions in the developed countries where the samples and data are sent for further analyses and tests [15]. Though collected from research participants by researchers in the academic and research institutions, samples are considered as the property of governments where the research participants who provide the samples come from and research ethics committees (RECs) are mandated with the task of reviewing the MTAs on behalf of their institutions. Some of the RECs are institutional reviews boards (IRBs) based in the academic or research institutions that send the samples and data while others are government-owned RECs that belong to research units of the Ministries of Health (MoH). These RECs are considered as custodians of the samples/data and they are seen to represent the interests of research participants who provide the samples/data. Today, completion of the MTA forms has become standard practice and it is intended to discourage researchers in academic and research institutions from shipping samples and data to institutions in developed countries and to encourage them to develop local capacity and expertise to perform all tests and analyses of samples and data in institutional labs in the developing countries. The completed MTAs have to be reviewed and approved by REC members before the samples and data are exported. The MTAs are one feasible and fair mechanism to prevent abuse and exploitation of human samples and data for clinical research while promoting autonomy of research participants through informed consent. Most MTAs address the following issues [11]:

- Intention for collecting the sample/data.
- Justification for requiring a biological sample/data.
- Duration for storage of the sample/data and its justification.
- The place where the sample/data will be kept.
- Ownership
- Control of the sample/data.
- Access

However, there are variations in regulations governing use of biological samples and data in Sub-Saharan Africa. The National Health Act of 2003 of South Africa requires that research participants must provide informed consent before donating biological samples and data but the Act does not have guidelines on reuse of such samples and data in research [7]. The Standard Operating Procedures for Biomedical Research in Botswana require prospective donors to be provided with the options to decide whether their donation should be stored for future use and that the Health Research and Development Committee (HRDC) must approve any protocols that would reuse the tissue samples and data [7]. The Gambian guidelines developed in 2001 do not require informed consent for anonymized unlinked samples, but regulatory approval of the government or the Ethics Committee is required for the reuse of the anonymized samples and data [7]. However, the Gambian guidelines require researchers to obtain informed consent from research participants for all identifiable samples and data but this may be waived by the Ethics Committee [6]. The Ugandan regulations require that a consent form be provided in order to collect and store a biological sample, in addition to providing information on storage, study purpose, confidentiality and future use. In the Ugandan case, research participants have the option of deciding whether their samples are stored to be used in the future or not [7]. In Zambia, the 2013 regulations state that “biological material and data can only be collected for the manner in which it was indicated in the research protocol” [7] but the regulations do not allow secondary use of biological samples and data. In Malawi, the research ethics regulations and guidelines do not allow the use of stored biological samples and data in future research [8].

The variations in national regulations and guidelines reflect the differing perspectives of research participants across several African countries. A study done in Uganda (*n* = 343) found that 95 % of the research participants would consent to reuse of samples and data without additional consent as long as the Institutional Review Board approved it [6]. Another study done in Egypt (*n* = 600), 44.3 % of the participants felt consent forms should include a separate section for storage and future use of samples [7]. In a recent South African study (*n* = 200), 77.5 % of the participants were comfortable with storage of samples while 12 % of the participants said they would require reasons for storage and would want to give separate consent for storage [7]. In a study conducted in Kenya by Langat, it was reported that a large proportion of proposals submitted to ethics committees requested the re-use of samples either in archives or from previous research projects but half of the consent forms of these protocols did not provide information to potential research participants nor did they request permission from research participants about either storage or exportation of their samples [16]. This diversity comes as no surprise as Africa is a multicultural society and the views of one country may not be indicative of the rest of the continent [7]. To the best of our knowledge, there is no empirical study conducted in Malawi that assessed the view of research participants and community members with regards to storage and future use of biological samples and data.

## Benefit/risk assessment of current practices in Malawi

Currently, research ethics committees in Malawi do not allow storage and use of biological samples and data for future unspecified research [4, 5]. The National Commission for Science and Technology (NCST) in a document titled: What is the National Regulatory Requirement and Position on Accessing, Collection, Storage and use of Human Biological Specimen for research in Malawi stipulates that researchers are not allowed to collect biological specimens and data that are not required to address the study objectives; tests on biological specimens should only be as approved by the approved protocol and specimens collected for a particular purpose should not be used for any other purposes [4, 5]. The NCST document further states that it is not allowed in Malawi to consent research participants to collection, use and storage of specimens for future use in research or other purposes [4, 5]. This stand has been adopted and is being implemented by the local research ethics committees such as the National Health Sciences Research Committee (NHSRC) and the College of Medicine Research and Ethics Committee (COMREC) [9].

However, Malawi has benefited greatly from research done on stored tissue, blood samples and data. In 2001, Glynn et al. published the results of a study which showed that HIV had been present in Karonga (northern Malawi) as early as 1982 [12]. The samples and data that were used had been collected for a different purpose in a population survey dating back to 1981 [12]. Reuse of these samples and data was authorized by the NHSRC.

Malawi like other developing countries has limited capacity to conduct complex biomedical and genetic tests required to answer scientific and ethically valid questions. This has allowed researchers to obtain ethics approval from local ethics committees to export biological samples and data to countries where such biomedical and genetic tests are available. However, the regulatory authorities are concerned that these exported biological samples and data may be subject to abuse and exploitation by researchers. Indeed, international collaborative research has been subject to abuse and exploitation in the past and not only on issues of stored biological material [10]. There has been differential exposure to risks among communities in developing countries as compared to their counterparts in the developed world and lack of or unequal access to benefits as well as potential profits from research [10]. This is expressed in the views of some research participants with regard to benefit sharing. In a South African study (*n* = 200), over a third (39.5 %) of participants indicated that “they would mind if researchers or research organizations generated profits from the research in which they were involved” [7]. Of this group, 43 % would want a share of the profits [7].

It should also be noted that while it is the duty of government and RECs to promote the interests of the research participants and prevent their exploitation, such efforts may amount to unnecessary paternalism if the affected research participants are not involved in such decisions [10]. For examples, the restriction placed on consenting research participants not to donate biological samples and data for future research in the NCST document can be interpreted to assume that research participants in Malawi are not capable of deciding on their own how biological samples and data collected from them may be used. This would be against the principle of autonomy or respect for persons.

John Rawls, one of the contemporary philosophers once stated that "justice as fairness serves as a basis of informed and willing political agreement between citizens viewed as free and equal persons “[13]. Thus, when justice is taken for fairness, it is important to bear in mind that there are many ways of being fair and being ethical. Hence, the NCST seems to suggest that not allowing the secondary use of biological samples and data is the only way to avoid doing wrong and yet there are more important factors that come prior to the collection of biological samples and data that should matter most. Such factors include the respect of autonomy through ensuring that informed consent is obtained when collecting biological samples and data for any future use. It would be fair to allow competent research participants to make such decisions on their own without being paternalistic.

Restrictions on secondary use of biological samples and data imply that any tissue remaining at the completion of a particular study should either be kept in storage indefinitely or be disposed of. This amounts to wastage of valuable biological material that could be used to advance medical knowledge for better interventions and such a restriction is not cost effective. This restriction also subjects research participants to biological sample and data recollection in order to carry out secondary research. This exposes them to additional risk and contradicts the principle of non-maleficence.

The restrictions set forth by Malawian regulations and guidelines also run the risk of selective justice which is another form of injustice. While being restrictive on biological samples and data derived from research, which is heavily controlled and closely monitored by research ethics committees, regulatory bodies are silent on samples and data derived from medical centers for clinical diagnosis that could end up in research centers. Therefore, we propose the introduction and utilization of a separate material transfer agreement which shall be called medical material transfer agreement (MMTA) for exportation of biological samples and data that are collected from patients in both private and public health facilities for clinical purposes.

Both the National Health Sciences Research Committee and COMREC do require completion and approval of MTAs for biological samples and data collected in research settings. However, there is no data and material transfer agreement that is designed for biological samples and data collected in clinical settings that may later on be used for research purposes. This may create a gap for exploitation of biological samples and data collected for diagnostic purposes. Although comprehensive, the MTA in research settings does not address the issue of how samples and data will be handled after research activities are over. As such, there is still room for researchers to exploit biological samples and data collected for research purposes. Creating a bio-bank or bio-repository center in the country and requesting return of samples and data after a specified period may be a viable alternative to better regulate handling of human samples and data for medical research.

However, we are of the opinion that broad consent for storage and future use of biological samples and data done at the time of sample and data collection may be the best model of consent to allow unspecified ethical research that will be reviewed and approved by an ethics committee [14]. By definition, broad consent is “consent for an unspecified range of future research subject to ongoing research oversight [18]. By giving broad consent, a research participant is deemed to have surrendered control of their biological sample and data and implicitly agreed to use of the sample and data in future studies as long as the studies are reviewed and approved by research ethics committees and there is a possibility to withdraw consent [3]. This means that individual research participants who provide broad consent are protected by research oversight mechanisms which ensure that future proposed research is ethical and poses no greater than minimal risk to the research participants and the research participants themselves have the right to withdraw their samples and data. In addition, Grady and colleagues have reported on empirical studies that have showed that individual research participants who provide broad consent are reassured that their interests will be protected when oversight mechanisms are in place to review future proposed research [18, 19]. Furthermore, in an analysis of informed consent documents for 13 of the 19 H3 Africa projects done by Munung and others, they reported that 8 of the H3 projects used broad consent while 4 research projects used tiered consent and 1 used specific consent [20]. This means that broad consent is still the most preferable model of consent used in genetic and genomic studies in Africa. Hence, broad consent may address the problem of giving consent for future use of previously collected samples and data.

There are also other consent models which could be used. A multi-layered/tiered consent is a consent model in which research participants are given a set of options which allow them to select how they want to participate in the research. Multi-layered/tiered consent can used where researchers want to give options to research participants to choose whether they want their samples and data to be used in future studies or not [21]. This empowers research participants to consent to use of their samples and data for a particular study and in selected future related studies. However, tiered consent does not allow researchers to use research participants’ samples and data for research in which the research participants did not consent to [21]. Finally, researchers can choose to obtain specific consent from research participants in which case the research participants will permit the use of their biological samples and data just for the current study and they will not allow any use of their samples and data in future studies [1, 7]. Our understanding is that it is possible to obtain ‘informed consent’ from research participants using the three different models of consent we have highlighted above as long as potential research participants are provided with adequate information, they have understood that information and after considering the information, they have arrived at decisions to participate in research or not without having been subjected to coercion, undue influence, inducement or intimidation.

In circumstances where the research ethics committee recommends re-consenting of research participants having obtained specific consent in primary research projects, researchers would then be obliged to do so but if it is not feasible to re-contact research participants for consent to use stored biological samples and data for other purposes, the research ethics committee may provide a waiver or may consent on behalf of the research participants. As Nienaber puts it, “research in vulnerable populations is not by definition exploitative and unethical” and “paternalistic attitudes are denigrating” [10]. Therefore, measures taken to safeguard research participants from exploitation need to be reasonable, not very restrictive and should be respectable of the population being protected. After all, as Tindana and others have noted, “consent processes are a means of respecting research participants’ autonomy and decision-making capacity as well as enabling them to choose to avoid any potential harms of research by declining to take part in research” [17]. As such, paternalistic tendencies to safeguard exploitation of research participants are unreasonable and they do not respect the autonomy of consenting research participants.

## Recommendations

The current view held by the National Health Sciences Research Committee and COMREC are restrictive and may prove to be detrimental to health research in Malawi. As such, there is need for review of the policy stand at the national level. Research participants must be allowed to give informed consent if they so wish or to decline use of their biological samples and data derived from them for any other purpose including in future research. As an important component of medical research, research participants’ autonomy should be respected by allowing them to decide what they feel is the best fate for their donated biological samples and data. This could either be biological sample/data reuse or destruction.

Research ethics committees and regulatory bodies should continue doing vibrant reviews of medical research and put in place measures to avoid research participants’ exploitation and abuse. To better address regulatory bodies’ concern about exploitation of biological samples and data by researchers, further empirical social science research needs to be conducted locally to assess the views of research participants and the public on collection, storage and future reuse of biological samples and data and inform policy makers accordingly. There is also need to educate the public about the benefits of future use of biological samples and data.

We also recommend that government and local research institutions should invest in developing infrastructure that will allow processing of biological samples and data locally. In the absence of local capacity, it is unfair to hinder exportation of biological samples and data for good biomedical research. This would allow better regulation of biological samples and data that are collected both for medical research and diagnostic purposes.

There is also need to introduce a medical data and material transfer agreement document for biological samples and data derived in medical centers for clinical diagnostic purposes that could end up in research centers. The current policy is rigid and too strict on research derived samples and data but there is no strict control on biological samples and data derived from clinical investigations or procedures.

The current framework is also silent on proof of destruction of biological samples and data when the research is over. Hence, we recommend the development of a local bio-bank in Malawi where biological samples and data should be returned after the agreed period of storage has passed. Since establishing a bio-bank is likely to take time, a remote site can keep the biological samples and data in storage while allowing Malawian researchers the right to access the biological samples and data.

## Conclusions

As genetic, genomic and biomedical research technologies become more advanced, biological samples and data collected for specific research objectives may be of great value to study other secondary outcomes. Where genetic and genomic studies are involved, we are aware that the National Health Sciences Research Committee is mandated to review them. However, in both clinical research and genetic/genomic studies, much emphasis should be placed on collection, storage and obtaining consent for future reuse of biological samples and data. This has the potential to greatly benefit the nation and the medical research community in Malawi. However, this can only occur if the guidelines governing such research are reasonable, fair and objective. It is therefore necessary to review existing guidelines and allow secondary use of stored biological samples and data. Asking research participants to donate a sample or data for each research question when new research questions could potentially be answered by already existing samples and data is unnecessary duplication and unfair. This underutilization of biological samples and data constitutes reckless wastage of valuable and scarce human biological samples and data. While we acknowledge the genuine ethical concern by the National Commission for Science and Technology that it is trying to avoid exploitation of biological samples and data collected in Malawi for research purposes, there is no empirical evidence that researchers can exploit samples and data they collect in primary research projects. In addition, the current regulatory framework is punitive to those who want to be efficient for other emerging research questions. Too much restriction may also undermine training of local researchers as most high technology research is currently conducted outside the country.

## Abbreviations

ASHG: American Society of Human Genetics
CFR: Code of Federal Regulations
COMREC: College of Medicine Research and Ethics Committee
DTA: Data Transfer Agreement
HIV: Human Immuno-deficiency Virus
HRDC: Health Research and Development Committee
IRB: Institutional Review Board
MMTA: Medical Material Transfer Agreement
MTA: Material Transfer Agreement
NBAC: National Bioethics Advisory Commission
NCST: National Commission for Science and Technology
NHSRC: National Health Sciences Research Committee
US: United States of America
